# ICI 182,780 (Fulvestrant™) – the first oestrogen receptor down-regulator – current clinical data

**DOI:** 10.1054/bjoc.2001.1982

**Published:** 2001-02-01

**Authors:** J F R Robertson

**Affiliations:** Department of Surgery, City Hospital Nottingham, Nottingham NG5 IPB, UK

**Keywords:** ICI 182,780, anastrozole, tamoxifen, antioestrogens, advanced breast cancer, adjuvant therapy

## Abstract

ICI 182,780 (Fulvestrant™) is the first in a new class of novel, steroidal, ’pure‘ antioestrogens – the oestrogen receptor (ER) down-regulators. Its unique mode of action and the absence of partial agonist activity make it a candidate for the treatment of advanced breast cancer in both pre- and postmenopausal women. Tamoxifen has been available for use over the past 25 years. However, its partial agonist activity has been associated with detrimental effects, particularly on the endometrium, and may be associated with the development of tamoxifen resistance. Other antioestrogen agents have previously been unable to demonstrate clinically relevant activity following the development of resistance to tamoxifen. In contrast, the unique mechanism of action of ICI 182,780 results in significant clinical activity in patients failing on tamoxifen therapy. Indeed, phase III clinical trials have demonstrated that ICI 182,780 is at least as effective as the aromatase inhibitor anastrozole in the treatment of postmenopausal patients with advanced disease who have progressed during threatment with prior enocrine therapy. As such, ICI 182,780 will provide a valuable addition to the armamentarium for the treatment of advanced breast cancer. © 2001 Cancer Research Society


*British Journal of Cancer* (2001) **85**(Suppl 2), 11–14
© 2001 Cancer Research Campaign
doi: 10.1054/ bjoc.2001.1982, available online at http://www.idealibrary.com on IDEAL

### ICI 182,780 (Fulvestrant™) – the first oestrogen receptor down-regulator – current clinical data

**JFR Robertson**

Department of Surgery, City Hospital Nottingham, Nottingham NG5 IPB, UK

**Summary** ICI 182,780 (Fulvestrant™) is the first in a new class of novel, steroidal, 'pure' antioestrogens – the oestrogen receptor (ER) down-regulators. Its unique mode of action and the absence of partial agonist activity make it a candidate for the treatment of advanced breast cancer in both pre- and postmenopausal women. Tamoxifen has been available for use over the past 25 years. However, its partial agonist activity has been associated with detrimental effects, particularly on the endometrium, and may be associated with the development of tamoxifen resistance. Other antioestrogen agents have previously been unable to demonstrate clinically relevant activity following the development of resistance to tamoxifen. In contrast, the unique mechanism of action of ICI 182,780 results in significant clinical activity in patients failing on tamoxifen therapy. Indeed, phase III clinical trials have demonstrated that ICI 182,780 is at least as effective as the aromatase inhibitor anastrozole in the treatment of postmenopausal patients with advanced disease who have progressed during threatment with prior enocrine therapy. As such, ICI 182,780 will provide a valuable addition to the armamentarium for the treatment of advanced breast cancer. © 2001 Cancer Research Society

**Keywords**: ICI 182,780; anastrozole; tamoxifen; antioestrogens; advanced breast cancer; adjuvant therapy

### INTRODUCTION

The development of effective endocrine therapies for the management of early and advanced breast cancer has for a long time focused on modifying the response of hormone-sensitive tumours to circulating oestrogens. In theory, this endocrine manipulation can be performed at a variety of levels: altering circulating levels of oestrogens, modifying ligand binding to the oestrogen receptor (ER) and adapting downstream responses as a consequence of ligand binding to the ER.

These differing approaches have led to the development of the most widely used agents, the nonsteroidal antioestrogens – alternatively described as selective oestrogen receptor modulators (SERMs) (Kauffman and Bryant, 1995). Much of our clinical experience of antioestrogens has been gleaned from the use of tamoxifen (Nolvadex™). Tamoxifen has been available for more than 25 years (Early Breast Cancer Trialists' Collaborative Group, 1992) and during this period has become established as the first-choice endocrine therapy for the management of postmenopausal women with hormone-sensitive early and advanced breast cancer. Recent clinical evidence has shown that tamoxifen also reduces the risk of breast cancer (Fisher et al, 1998), on the basis of which it is now licensed in the USA for use in patients considered to be at high risk of developing primary breast cancer.

The clinical experience of tamoxifen is very extensive with the equivalent of about 8 million patient years of exposure during the last 25 years. In the adjuvant setting, tamoxifen treatment provides significant reductions to both tumour recurrence rates and overall mortality (Early Breast Cancer Trialists' Collaborative Group, 1998; see Baum, p 15). However, despite these benefits, there remain a number of significant clinical issues which may limit tamoxifen's long-term use.

Many of these concerns have centred primarily on tamoxifen's partial agonist activity. While offering benefits to bone mineral density and blood lipid profiles, this agonistic activity has also been associated with detrimental effects upon the endometrium (Assikis and Jordan, 1995) and increases the risk of thromboembolic disease in some patients. Furthermore, it is thought that the development of tamoxifen resistance necessitating furthe therapies is due, in part, to this partial agonist activity (Plotkin et al, 1978; Howell et al, 1995a).

Consequently, success in identifying an improved endocrine agent is reliant upon resolving these negative trophic effects, while maintaining or perhaps improving on the advantages that tamoxifen offers. To achieve this end, recent attention has focused on the third-generation aromatase inhibitors, including anastrozole ('Arimidex™'), which exert their clinical effects by blocking the peripheral production of oestrogens and thus significantly impact on total circulating oestrogens. The aromatase enzyme converts androgen substrates to oestrogen, the primary source of the hormone in postmenopausal women. However, aromatase inhibitors cannot abrogate the trophic actions of dietary or environmental oestrogens.

A third class of agents currently attracting significant clinical interest are the oestrogen receptor (ER) down-regulators, which act by completely suppressing the effects of oestrogens as a consequence of disrupted nuclear localization of ligand–ER complexes and increased proteolytic degradation of the ER (Howell et al, 2000a). ICI 182,780 (Fulvestrant™) is the first agent to be identified in this class and may represent an important advance in endocrine therapy. Acting as a 'pure' antioestrogen, ICI 182,780 lacks any partial agonist activity. Consequently, it is expected that ICI 182,780 may offer a longer duration of treatment response with a decreased risk of thromboembolism and the unwanted endometrial side-effects that have been associated with tamoxifen treatment in some patients. ICI 182,780 is currently being clinically evaluated in global phase III trials.

### OESTROGEN RECEPTOR DOWN-REGULATION – A NOVEL MODE OF ACTION

The majority of antioestrogens that have been identified to date, including tamoxifen, are predominantly nonsteroidal in structure. In contrast, ICI 182,780 is an oestrogen analogue compound with an alkylsulphinyl side-chain at position 7α (Figure 1). This compound was identified in the late 1980s in a systematic programme to identify agents which completely blocked oestradiol-stimulated uterine proliferation in the rat without stimulating proliferation in the absence of oestrogen, indicating a pure antioestrogenic activity (Wakeling et al, 1991). ICI 182,780 as been in clinical development since the early 1990s.

In addition to structural distinctions, ICI 182,780 also differs from tamoxifen in its novel mode of action. Although both agents mediate their clinical effects through the ER, they differ significantly in the downstream sequence of events after binding. Oestradiol, the natural ligand for the ER, binds and stimulates receptor dimerization and subsequent activation of two activation domains (AF1 and AF2, Figure 2). The activated receptor complex then undergoes nuclear localization and subsequent binding to discrete DNA sequences – oestrogen response elements (EREs) – located in the upstream regulatory regions of oestrogen-regulated genes. Upon binding to these sequences, transcriptional coactivators are recruited to the complex, resulting in activation of gene expression. In contrast, tamoxifen binds to the ER and dimerization occurs but fails to activate the AF2 domain (Figure 2). Ultimately, this causes incomplete attenuation of transcription and thus, while tamoxifen exerts antioestrogen activity, the active AF1 domain allows a degree of gene expression to occur, resulting in tamoxifen's partial agonist activity. In contrast, while ICI 182,780 is capable of binding to the ER, receptor dimerization is impaired, receptor degradation is accelerated by increase of receptor protein turnover, and AF1 and AF2 remain inactive. Ultimately, this results in disrupted nuclear localization and a failure to recruit transcriptional coactivators. Consequently, ER-mediated transcription is completely attenuated, leading to complete suppression of the expression of oestrogen-dependent genes, and thus ICI 182,780 can be described as a 'pure antioestrogen' (Wakeling, 1995).

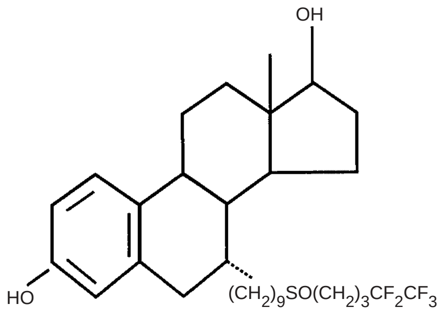

**Figure 1** ICI 182,780, a steroidal oestrogen receptor down-regulator

### PRECLINICAL DATA

Early evidence from preclinical animal models provided the first data indicating that ICI 182,780 may offer a potential therapeutic advance over tamoxifen. In addition, these studies have also provided strong supporting evidence for ICI 182,780's unique mechanism of action. Early observations from oestrogen receptors in the rat uterus demonstrated that ICI 182,780 has a greater binding affinity for the ER than tamoxifen (Wakeling et al, 1991). In vivo models have shown that ICI 182,780 is effective in inhibiting the growth of tamoxifen-resistant (Coopman et al, 1994) and tamoxifen-stimulated cell lines (Osborne et al, 1994). In nude mice xenografted with MCF-7 tumour cells, superior efficacy of ICI 182,780 was demonstrated by showing a near-doubling of the mean duration of tumour control compared with both tamoxifen and oestrogen withdrawal (Figure 3) (Osborne et al, 1995). Moreover, some of the tumours examined in this study did not re-grow, even 2 years after initial follow-up. Animal models have

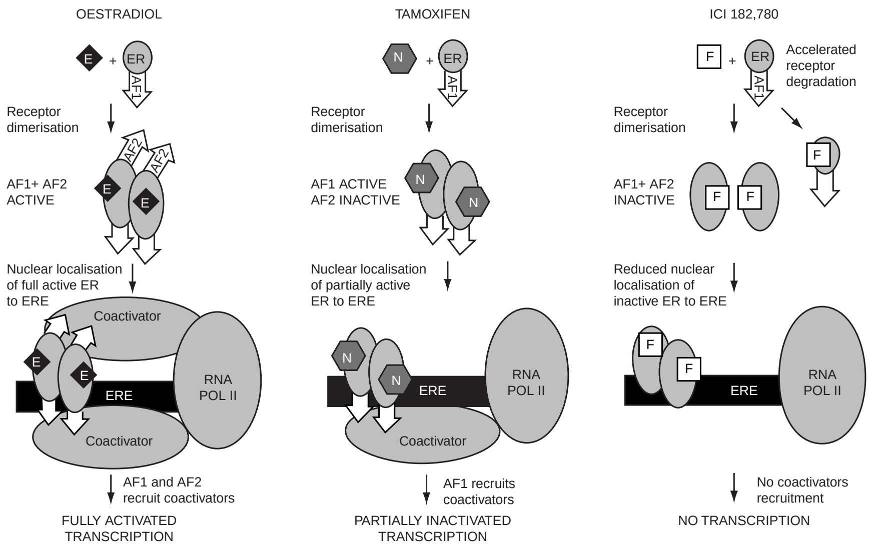

**Figure 2** Mechanisms of action – oestradiol, tamoxifen and ICI 182,780. Each antioestrogen differs in the mode of action at the level of the oestrogen receptor (ER). Molecular events that normally occur include receptor dimerization and binding to discrete oestrogen-response elements in oestrogen-regulated genes, followed by recruitment of other proteins to form a transcriptional complex, leading to either transcriptional activation or repression (adapted with kind permission from the Society of Endocrinology from Wakeling 2000) ER = oestrogen receptor, ERE = oestrogen-response elements, AF = activation function, RNA POL II = RNA polymerase II, E = oestradiol, N = Nolvadex (tamoxifen), F = Fulvestrant (ICI 182,780)

 *© 2001 Cancer Research Campaign*

also shown that ICI 182,780 probably does not cross the blood–brain barrier (Wade et al, 1993), thereby potentially minimizing hot flushes in patients. Furthermore, these models have demonstrated that not only does ICI 182,780 have no proliferative effect on the uterus, but it also blocks tamoxifen's uterotrophic activity (Wakeling et al, 1991).

### CLINICAL DATA

### Presurgical trials

A large-scale trial of patients scheduled to undergo surgery for removal of primary breast tumours compared the antitumour effects of ICI 182,780 with those of tamoxifen (Robertson et al, 2000). A total of 200 postmenopausal women with primary breast cancer were randomized to receive a range of doses of ICI 182,780 (50–250 mg) administered intramuscularly, or a daily oral dose of tamoxifen (20 mg/day) or matching tamoxifen placebo for 14–21 days prior to surgery.

This study demonstrated a clear dose-dependent reduction in the levels of ER expression, as determined by the ER index. The reduction in ER index was significantly different from placebo at the three doses of ICI 182,780 administered. When the clinically used dose of ICI 182,780 (250 mg) was compared with tamoxifen, a significantly greater reduction in the ER index was observed ($P = 0.02$), indicating greater activity for ICI 182,780. All doses of ICI 182,780 significantly reduced progesterone receptor (PgR) expression – an oestrogen-regulated gene – in a dose-dependent manner. Compared with placebo, the two higher doses of ICI 182,780 (125 and 250 mg) significantly reduced PgR expression. All three doses of ICI 182,780 reduced progesterone receptor expression to a greater extent than tamoxifen, which actually resulted in a stimulation of PgR expression. Ki67 levels, a cell cycle marker of proliferation, were significantly reduced at all three ICI 182,780 doses (50, 125 and 250 mg) compared with placebo, suggesting a potent antiproliferative effect of ICI 182,780.

This is the first clinical evidence in human breast cancer consistent with preclinical data that also supported the hypothesis of two distinct mechanisms of action for the two agents. This study also offers direct evidence in support of the concept that ICI 182,780 is

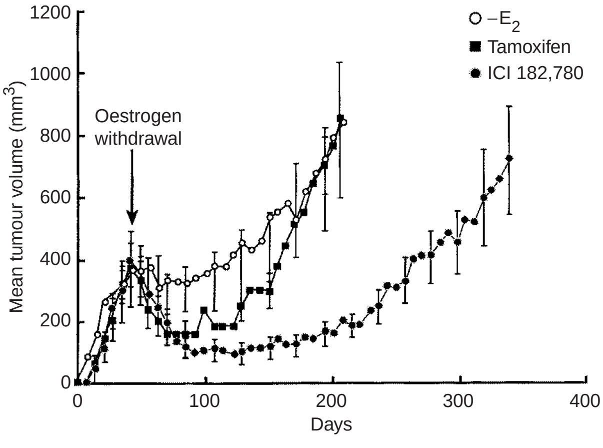

**Figure 3** Effects of oestrogen withdrawal, tamoxifen and ICI 182,780 on MCF-7 tumour growth (adapted with kind permission from Oxford University Press from Osborne et al, 1995)

a functional ER down-regulator in vivo and that it provides significant and potent antioestrogenic and antiproliferative activities.

The clinical efficacy of ICI 182,780 has been observed in other clinical trials. A phase II study was conducted involving 19 postmenopausal women with advanced breast cancer whose disease had progressed after prior treatment with tamoxifen. In total 69% of patients had tumour remission after treatment with 250 mg per month ICI 182,780 with either an objective response (OR) or stable disease for more than 24 weeks (SD ≥ 24 weeks). In this group of patients, the median duration of objective response was 26 months (Howell et al, 1995b; Robertson et al, 1997). In addition, this initial trial provided important evidence that ICI 182,780 was well tolerated with no increase in deaths, withdrawals or significant serious adverse events reported. Thus, this trial clearly demonstrated absence of cross-resistance between tamoxifen and ICI 182,780, supporting observations made in the earlier preclinical studies. This observation adds further evidence distinguishing ICI 182,780 from other antioestrogens.

### ICI 182,780 phase III trials

The early presurgical and phase II clinical trials supported the initiation of a major phase III clinical programme in postmenopausal women with advanced breast cancer whose disease had progressed during or after prior endocrine therapy (the majority of patients (98%) had received prior tamoxifen treatment). Two phase III, randomized, parallel-group studies (North American (NA; $n = 400$) (Osborne et al, 2001) and rest of the world (ROW; $n = 451$) (Howell et al, 2000b)) compared the efficacy and tolerability of 250 mg of ICI 182,780 with the aromatase inhibitor anastrozole, 1 mg, in the treatment of advanced breast cancer. In essence, these trials compared the efficacy of an antioestrogen (ICI 182,780) administered after the failure of another antioestrogen (tamoxifen) with the efficacy of an aromatase inhibitor (anastrozole) given after an antioestrogen. This is particularly interesting given the recent observation that demonstrates a superior efficacy of anastrozole compared with tamoxifen in the treatment of advanced disease. Anastrozole was the first aromatase inhibitor to be licensed for first-line treatment in advanced breast cancer (see Buzdar, p 6).

Both trials were virtually identical in their design. However, there were some notable differences between the two. Firstly, the ROW trial had an open, non-blinded design while the NA study was performed under double-blind, double-dummy conditions (by employing an ICI 182,780 placebo injection in the anastrozole group and placebo anastrozole tablets in the ICI 182,780 group). Secondly, the NA trial delivered two 2.5 ml injections of ICI 182,780, while the ROW trial employed only a single 5 ml injection.

Time to progression (TTP), the primary efficacy end-point, was not significantly different between the ICI 182,780 and anastrozole arms of both trials (NA trial: median TTP 5.4 months vs 3.4 months, HR 0.92 (0.74–1.14) $P = 0.43$; ROW: 167 days vs 156 days, HR 0.98 (0.80, 1.21), $P = 0.84$). The OR (complete response (CR) + partial response (PR) rate in the ROW trial was 20.7% in the ICI 182,780 group compared with the anastrozole arm (15.7%, $P = 0.20$). Clinical benefit (CR + PR + SD ≥ 24 weeks) was similar in both treatment arms (approximately 45%). In the NA trial the OR was identical in both treatment arms (17.5%, $P = 0.96$) and there was a 5% greater clinical benefit with ICI 182,780 (42%) compared with anastrozole (36%).

*© 2001 Cancer Research Campaign* 

When specific consideration was given only to those patients who had responded to endocrine treatment, a near-doubling of tumour response time was observed in the double-blind NA trial (median duration of response (DOR) 19.3 months with ICI 182,780 vs 10.5 months with anastrozole in the NA trial). In the ROW trial a similar median DOR for both treatments was observed (434 days vs 425 days). In summary, ICI 182,780 was at least as effective as anastrozole for all major primary and secondary efficacy end-points in the management of advanced breast cancer.

Data from the NA and ROW studies indicate that both ICI 182,780 and anastrozole are generally well tolerated in these women, which is particularly encouraging given the longer clinical experience with anastrozole. Similar numbers of patients in both studies withdrew due to an adverse event (3.2% vs 2.2% for ICI 182,780 and anastrozole in the ROW trial; 2.5% vs 2.6% in the NA trial). In the ROW trial, predefined adverse events for ICI 182,780 and anastrozole included: hot flushes (18.6% vs 17%), gastrointestinal disturbances (40.0% vs 34.3%), weight gain (0.5% vs 1.7%) and vaginitis (0.5% vs 0.9%). In the NA trial, these predefined events were: hot flushes (23.5% vs 24.9%), gastrointestinal disturbances (53.4% vs 56.0%), weight gain (1.5% vs 1.6%) and vaginitis (3.4% vs 2.6%). Data from both trials indicate that the injection of ICI 182,780 was well tolerated.

### CONCLUSION

Currently, tamoxifen is the most widely used agent for the treatment of advanced breast cancer. While aromatase inhibitors offer further clinical benefit following tamoxifen failure, alternative antioestrogens offer little if any clinical benefit. In contrast, the ER downregulator ICI 182,780, is highly effective in the treatment of postmenopausal patients with advanced breast cancer who have progressed on prior endocrine therapy. Indeed the clinical trial programme indicates that ICI 182,780 is at least as effective as anastrozole. Given the efficacy and tolerability it has demonstrated so far, combined wiith the convenience of a once-monthly intramuscular injection, ICI 182,780 provides a valuable addition to the currently available options for the treatment of advanced breast cancer.

### REFERENCES

Assikis VJ and Jordan VC (1995) Gynecologic effects of tamoxifen and the association with endometrial carcinoma. *Int J Gynecol Obstet* **49**: 241–257

Coopman P, Garcia M, Brunner N, Derocq D, Clarke R and Rochefort H (1994) Anti-proliferative and anti-estrogenic effects of ICI 164,384 and ICI 182,780 in 4-OH-tamoxifen-resistant human breast-cancer cells. *Int J Cancer* **56**: 295–300

Early Breast Cancer Trialists' Collaborative Group (1992) Systemic treatment of early breast cancer by hormonal, cytotoxic or immune therapy. 133 randomized trials involving 31,000 recurrences and 24,000 deaths among 75,000 women. *Lancet* **339**: 1–15

Early Breast Cancer Trialists' Collaborative Group (1998) Tamoxifen for early breast cancer: an overview of the randomized trials. *Lancet* **351**: 1451–1467

Fisher B, Costantino JP, Wickerham DL, Redmond CK, Kavanah M, Cronin WM, Vogel V, Robidoux A, Dimitrov N, Atkins J, Daly M, Wieand S, Tan-Chiu E, Ford L and Wolmark N (1998) Tamoxifen for prevention of breast cancer: report of the National Surgical Adjuvant Breast and Bowel Project P-1 Study. *J Natl Cancer Inst* **90**: 1371–1388

Howell A, DeFriend D and Anderson E (1995a) Clues to the mechanism of endocrine resistance from clinical studies in advanced disease. *Endocr Relat Cancer* **2**: 131–139

Howell A, DeFriend D, Robertson J, Blamey R and Walton P (1995b) Response to a specific antioestrogen (ICI 182780) in tamoxifen-resistant breast cancer. *Lancet* **345**: 29–30

Howell A, Osborne CK, Morris C and Wakeling AE (2000a) ICI 182,780 (Faslodex): development of a novel, 'pure' antiestrogen. *Cancer* **89**: 817–825

Howell A, Robertson JFR, Quaresma Albano J, Aschermannova A, Mauriac L, Kleeberg U, Vergote I, Erikstein B, Webster A and Morris C (2000b) Comparison of efficacy and tolerability of Faslodex™ (ICI 182,780) with Arimidex™ (anastrozole) in post-menopausal (PM) women with advanced breast cancer (ABC) – preliminary results. *Breast Cancer Res Treat* **64**: 27 (Abstr 6)

Kauffman RF and Bryant HU (1995) Selective estrogen receptor modulators. *Drug News Perspect* **8**: 531–539

Osborne CK, Jarman M, McCague R, Coronado EB, Hilsenbeck SG and Wakeling AE (1994) The importance of tamoxifen metabolism in tamoxifen-stimulated breast tumor growth. *Chemother Pharmacol* **34**: 89–95

Osborne CK, Coronado-Heinsohn EB, Hilsenbeck SG, McCue BL, Wakeling AE, McClelland RA, Manning DL and Nicholson RI (1995) Comparison of the effects of a pure steroidal antiestrogen with those of tamoxifen in a model of human breast cancer. *J Natl Cancer Inst* **57**: 746–750

Osborne CK, Pippen J, Jones SE, Parker LM, Ellis M, Come S, Gertler S, May JT, Burton G, Webster A, Morris C, Dimmery I and Elledge R (2001) 'Faslodex' (ICI 182,780) shows longer duration of response compared with 'Arimidex' (anastrozole) in post-menopausal (PM) women with advanced breast cancer (ABC). Preliminary results of a Phase III N. American trial (ABC) *Breast Cancer Res Treat* (**Abst**): *in press*

Plotkin D, Lechner JJ, Jung WE and Rosen PJ (1978) Tamoxifen flare in advanced breast cancer. *JAMA* **240**: 2644–2646

Robertson JFR, Howell A, DeFriend D, Blamey R and Walton P (1997) Duration of remission to ICI 182,780 compared to megestrol acetate in tamoxifen-resistant breast cancer. *Breast* **6**: 186–189

Robertson JFR, Nicholson RI, Anderson E, Dowsett M, Ellis I, Dixon JM, Bundred N, Webster A and Morris C (2000) The anti-tumor effects of single-dose, long-acting 'Faslodex' (ICI 182,780) compared with tamoxifen in post-menopausal primary breast cancer patients treated before surgery. *Breast Cancer Res Treat* **59**: 99

Wade GN, Blaustein JD, Gray JM and Meredith JM (1993) ICI 182,780: a pure antiestrogen that affects behaviors and energy balance in rats without acting in the brain. *Am J Physiol* **43**: R1392–R1398

Wakeling AE (1995) Use of pure antioestrogens to elucidate the mode of action of oestrogens. *Biochem Pharmacol* **49**: 1545–1549

Wakeling AE (2000) Similarities and distinctions in the mode of action of different classes of antioestrogens. *Endocr Relat Cancer* **7**: 17–28

Wakeling AE, Dukes M and Bowler J (1991) A potent specific pure antiestrogen with clinical potential. *Cancer Res* **51**: 3867–3873

 *© 2001 Cancer Research Campaign*
